# Standards bei der Anwendung der fiberendoskopischen Schluckuntersuchung in Deutschland: Eine Fragebogenerhebung

**DOI:** 10.1007/s00115-021-01127-8

**Published:** 2021-04-30

**Authors:** A. Wehner, B. Schumann-Werner, B. Fimm, B. Mall, C. J. Werner

**Affiliations:** 1 Klinik für Neurologie, Medizinische Fakultät, Uniklinik RWTH Aachen, Pauwelsstraße 30, 52074 Aachen, Deutschland; 2 Sektion Interdisziplinäre Geriatrie, Klinik für Neurologie, Medizinische Fakultät, Uniklinik RWTH Aachen, Aachen, Deutschland

**Keywords:** Neurogene Schluckstörungen, Dysphagiediagnostik, FEES, Standards, Befragung, Neurogenic dysphagia, Swallowing assessment, FEES, Standards, Survey

## Abstract

**Hintergrund:**

Die fiberendoskopische Schluckuntersuchung („fiberoptic endoscopic evaluation of swallowing“, FEES) gilt als ein unverzichtbares instrumentelles Verfahren im Management schluckgestörter Patienten. Das eingeführte Ausbildungscurriculum hat das Ziel, die Qualitätsstandards zu erhöhen und zu einer Aufwertung des Verfahrens beizutragen.

**Fragestellung:**

Die Studie untersucht, inwieweit eine standardisierte Durchführung, Auswertung und Dokumentation der FEES in Deutschland nach Einführung des Curriculums stattfindet.

**Material und Methoden:**

Insgesamt 603 neurologische und geriatrische Kliniken in Deutschland wurden mithilfe eines Onlinefragebogens bezüglich struktureller Merkmale und deren Ablauf der Untersuchung befragt.

**Ergebnisse:**

Insgesamt 190 Institutionen führten die Befragung vollständig durch. 43,31 % der Institutionen haben erst seit der Publikation des Curriculums die FEES implementiert. Die praktische Anwendung findet vermehrt durch Mediziner statt (59 %), das Schreiben des Befundes und die Kostempfehlung durch Logopäden (62 % und 83 %). Mit erhöhtem Ausbildungsgrad steigt die praktische Anwendung durch Logopäden. Die Durchführung weist trotz der Orientierung am Standardprotokoll nach Langmore besonders in Bezug auf die Durchführung der anatomisch-physiologischen Untersuchung, die verabreichten Konsistenzen und Nahrungsmittel und das Scoring schluckrelevanter Parameter Unterschiedlichkeiten auf.

**Diskussion:**

Die Einführung des Curriculums hat zur Aufwertung der FEES und zu einer Stärkung der Logopädie als durchführende Berufsgruppe geführt. Zum jetzigen Stand liegt ein in wesentlichen Aspekten homogener Ablauf der Untersuchung vor, der jedoch Bedarf nach weiterer Vereinheitlichung zeigt. Das FEES-Curriculum könnte als Steuerungsinstrument zur weiteren Standardisierung verwendet werden.

## Abstract (en)

**Background:**

The fiberoptic endoscopic evaluation of swallowing (FEES) is considered to be an indispensable instrumental procedure in the management of patients with dysphagia. The aim of the implemented training curriculum is to raise the quality standards and to contribute to an upgrading of the procedure.

**Objective:**

The study evaluated to what extent a standardized implementation, evaluation and documentation of FEES takes place in Germany after the introduction of the curriculum.

**Material and methods:**

In this study 603 neurological and geriatric hospitals in Germany were interviewed by the use of an online questionnaire regarding structural features and the course of the investigation.

**Results:**

A total of 190 institutions completed the survey. Of the institutions 43.31% had only implemented FEES since the publication of the curriculum. The practical application is increasingly carried out by physicians (59%), the clinical reports and cost recommendations are carried out by speech therapists (62% and 83%, respectively). The practical application by speech therapists increases with increasing level of training. Despite orientation towards the standard protocol according to Langmore, there are differences in the implementation of the anatomical physiological examination, the consistencies and foods administered and the scoring of swallowing-relevant parameters.

**Discussion:**

The introduction of the curriculum has led to an upgrading of the FEES and to a strengthening of speech therapy as the implementing professional group. At the current state of the art there is a homogeneous course of the examination in essential aspects but it shows a need for further uniformity. The FEES curriculum could be used as a guiding instrument for further standardization.

## Hintergrund

Neurogene Dysphagien stellen ein häufiges und gravierendes Symptom neurologischer Erkrankungen dar und sind mit schwerwiegenden funktionellen, psychologischen, sozialen und finanziellen Folgen verknüpft [1]. Die fiberendoskopische Schluckuntersuchung (FEES) als apparative Methode gilt als ein unverzichtbares Verfahren im Management schluckgestörter Patienten [2]. Die Durchführung richtet sich nach dem Standardprotokoll von Langmore [3]. Dennoch sind in der Literatur unterschiedliche Protokolle mit zahlreichen Variationen zu finden [4–6]. Die Auswertung lässt durch eine Vielzahl an Scores ebenfalls Unterschiedlichkeiten zu [7–10]. Die Popularität der Untersuchung hat in den letzten Jahren stark zugenommen, dennoch besteht weiterhin eine Forschungslücke im Hinblick auf die Standardisierung und Validierung des Verfahrens [11]. 2014 wurde von der Deutschen Schlaganfallgesellschaft (DSG) und der Deutschen Gesellschaft für Neurologie (DGN) ein Ausbildungscurriculum eingeführt mit dem Ziel, die Qualitätsstandards zu erhöhen und zu einer Aufwertung des Verfahrens beizutragen [12]. Die im Folgenden beschriebene Onlineumfrage hatte den Anspruch zu erheben, inwieweit zum Zeitpunkt der Studiendurchführung eine einheitliche Durchführung, Auswertung und Dokumentation der FEES innerhalb Deutschlands stattfand. Außerdem sollte erfasst werden, ob Parameter, wie der Ausbildungsgrad einer Institution, die Art der Institution und deren Routine einen Einfluss auf die Durchführung und Auswertung zeigen.

## Methodik

Im Zeitraum von Dezember 2018 bis Januar 2019 fand eine deutschlandweite Onlinebefragung statt. Insgesamt wurden 603 neurologische und geriatrische Akuthäuser und Rehakliniken eingeschlossen. Es handelte sich um eine personalisierte Befragung mit individuellem Zugangslink. Zu diesem Zeitpunkt bestand kein Wissen darüber, ob die Institutionen die Möglichkeit zur FEES-Anwendung in ihrem Haus besitzen. Der Fragebogen umfasste 70 Fragen und gliederte sich in folgende Abschnitte:

- Strukturmerkmale der Institution: Art und Standort der durchführenden Institution, deren Routine (Anzahl FEES-Durchführungen, Jahre FEES-Anwendung) sowie die an der FEES beteiligten Personen (Anzahl, Beruf, Ausbildungsgrad, Aufgabenbereich),
- Durchführungsschritte der Untersuchung in den Bereichen „vorbereitende Maßnahmen“, „anatomisch-physiologische Untersuchung“, „Schluckuntersuchung mit Nahrung“ und „Überprüfung therapeutischer Maßnahmen“,
- Befundung und Dokumentation: beurteilte Parameter in der Schluckuntersuchung und an den Institutionen verwendete Protokollbögen und Scores.

Durch eine bipolare, verbalisierte, 4‑stufige Antwortskala (nie: 1, gelegentlich: 2, häufig: 3, immer: 4) wurde die Häufigkeit der Durchführung von Untersuchungsschritten, beurteilten Aspekten und Scores untersucht. Dichotome und polytome Antwortformate wurden u. a. zur Erhebung der Strukturmerkmale, der eingesetzten Methoden und Scores verwendet.

Anschließend wurden Häufigkeiten errechnet und Lage und Streuungsmaße bestimmt. Um die Einheitlichkeit der Untersuchung in Deutschland zu bewerten, wurde ein inhaltlicher Algorithmus definiert. Dieser sah eine Unterteilung in drei Einheitlichkeitsgrade pro Untersuchungsschritt vor. Ein Untersuchungsschritt konnte als „einheitlich“, „ähnlich“ oder „unterschiedlich“ in seiner Durchführungshäufigkeit interpretiert werden. Bei den nominalskalierten Daten lagen zur Einteilung die Prozentwerte der durchführenden Institutionen zugrunde (einheitlich: > 90 oder < 10 %, unterschiedlich: 25–75 %, ähnlich: alle weiteren). Die Einheitlichkeitsgrade der ordinalskalierten Daten richteten sich nach der Streuung, in dem Fall nach dem Interquartilsabstand (einheitlich: IQA = 0, ähnlich: IQA = 1, unterschiedlich: IQA > 1). Zur Errechnung des Einflusses der Strukturparameter „Ausbildungsgrad“ und „Routine“ der Institution auf die Durchführung und Auswertung der Untersuchung wurden inferenzstatistische Verfahren eingesetzt. Hierbei wurden lediglich die Aspekte betrachtet, die sich in der vorhergehenden Analyse als heterogen herausgestellt haben.

## Ergebnisse

Von den 603 Kliniken, die in die Befragung eingeschlossen wurden, haben 190 den Fragebogen vollständig beantwortet (Rücklaufquote von 31,5 %). Für die Teilnehmer zeigt sich eine geographisch gleichmäßige Verteilung über die Postleitzahlbereiche in Deutschland ohne lokale Häufung.

### Strukturmerkmale der Institutionen

Insgesamt 42,63 % der Kliniken führen monatlich bis zu 10 Untersuchungen durch. Der Anteil an Kliniken mit 10 bis 40 monatlichen FEES ist mit 43,68 % ähnlich hoch. Mehr als 40 Endoskopien werden von 13,68 % durchgeführt.

Die Befragten wenden die Methode zwischen 1 und 25 Jahren in ihrer Institution an. Ein deutlicher Zuwachs an Kliniken ist in den letzten 5 Jahren beobachtbar. 43,31 % nennen 1 bis 5 Anwendungsjahre.

Durchschnittlich führen pro Institution 3,68 Personen die FEES durch (SD: 2,77). Nicht alle eingeschlossenen Kliniken haben einen Bezug zum FEES-Curriculum. In 28 % der Fälle ist niemand vor Ort, der das FEES-Curriculum durchlaufen hat. Im Schnitt haben 1,51 Personen pro Institution die Ausbildung absolviert (SD: 1,66). In 163 der Kliniken befinden sich FEES-Auszubildende (MW: 1,41; SD: 1,47). FEES-Ausbilder gibt es in 88 der teilgenommenen Einrichtungen (MW: 0,74; SD: 1,08).

**Figure 1:**
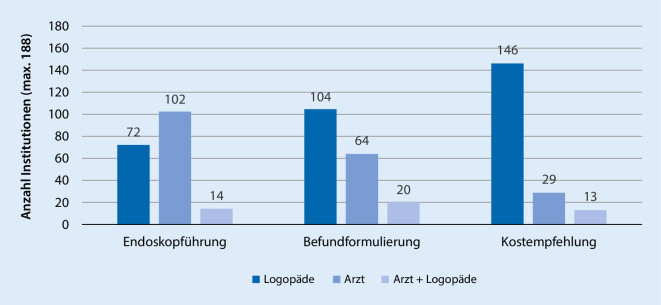

Für die Haupttätigkeiten bei der FEES-Durchführung (Endoskopführung, Befundformulierung, Kostempfehlung) werden hauptsächlich Ärzte und Logopäden angegeben. Die verantwortliche Disziplin unterscheidet sich jedoch zwischen den Aufgaben (Abb. 1). Die Endoskopführung findet zu 54,26 % allein durch Ärzte statt, zu 38,3 % ausschließlich durch Logopäden. Bei der Befundformulierung zeigt sich eine umgekehrte Verteilung (Logopäden: 55,32 %; Ärzte: 34,04 %). Dieses Bild verstärkt sich für das Aussprechen von Kostempfehlungen (Logopäden: 77,66 %; Ärzte: 15,43 %). Nur bei einem geringen Anteil an Kliniken (≤ 10 %) besteht keine klare Verantwortlichkeit und beide Berufsgruppen sind für die jeweiligen Aufgaben zuständig.

### Durchführung der FEES

Der Großteil der Einrichtungen führte regelmäßig eine *anatomische Untersuchung* (69 % „immer“, 17 % „häufig“) durch. Bei allen abgefragten Strukturen geben die meisten Institutionen an, diese in jeder Untersuchung zu betrachten. Die Werte für die Streuung sind gering. Folgende untersuchte Aspekte erhielten einen IQA von 0 und sind somit als einheitlich in ihrer Durchführungshäufigkeit zu betrachten: Strukturveränderungen, Form und Stellung der Stimmlippen, Aryknorpel und Epiglottis, Ansammlung von Speichel, Sekret und Speiseresten, Schlucken von Speichel und Reaktionen auf Retention, Penetration und Aspiration. Der Begutachtung respiratorischer und unwillkürlicher Bewegungen sowie der Schluckfrequenz ist ein IQA von 1 (= ähnlich) zugeordnet. Lediglich bei der Überprüfung des Sitzes einer Magensonde ist mit einem IQA von 2 (= unterschiedlich) eine hohe Streuung erkennbar.

Zur Beurteilung des Sekretmanagements geben 66,3 % an, einen Score zu verwenden (= unterschiedlich). Hierfür werden überwiegend folgende Scores eingesetzt: Sekretbeurteilungsskala von Murray ([9]; 68 Kliniken), Bogenhauser Dysphagiescore ([13]; 22 Kliniken) und Graduierung hypopharyngealer Speichelansammlungen nach Langmore ([3]; 11 Kliniken).

**Figure 2:**
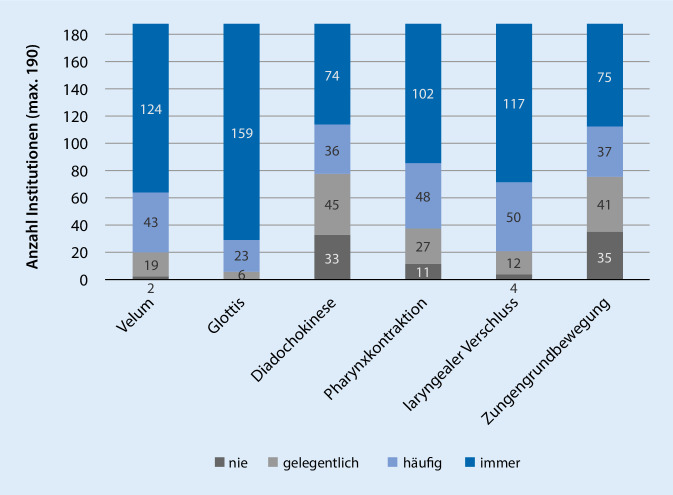

Die *motorische Funktionsprüfung* wird von 95 % der Institutionen regelmäßig durchgeführt (IQA 1 = ähnlich). Die Häufigkeitsverteilung der im Fragebogen thematisierten Parameter ist in Abb. 2 dargestellt. Bis auf die Überprüfung des Glottisschlusses zeigen alle Parameter eine mittlere bis starke Streuung in ihrer Durchführungshäufigkeit (IQA 1: velopharyngealer Verschluss, laryngealer Verschluss, Pharynxkontraktion; IQA 2: Diadochokinese, Zungengrundbewegung).

Die Häufigkeit der Durchführung einer *Sensibilitätsprüfung* streute stark (IQA 2 = unterschiedlich).

In der *Schluckuntersuchung mit verschiedenen Konsistenzen* werden Brei und Flüssigkeit von nahezu allen Kliniken in der FEES regelmäßig verabreicht (Brei: 99,47 %, Flüssigkeit: 98,95 %). Die Häufigkeit der beiden Konsistenzformen weist kaum Streuung auf (IQA 0 = einheitlich). Auch feste und angedickte Konsistenzformen werden von einem Großteil der Kliniken regelmäßig verwendet (fest: 87,3 %, angedickt: 79 %). Die Häufigkeit streute hierbei gering (IQA 1 = einheitlich). Die verabreichten Mengen zeigen sich für Flüssigkeit und Nektar bezogen auf die Überprüfung von Schlucken aus dem Glas und konsekutiven Schlucken unterschiedlich (Wasser: 80,53 % Schluck aus dem Glas, 64,74 % konsekutive Schlucke; Nektar: 67,8 % Schluck aus Glas, 52,5 % konsekutive Schlucke). Der Median für die Gesamtzahl verabreichter Boli liegt bei allen Konsistenzen auf der Stufe „2 bis 5 Schlucke“ pro Untersuchung (IQA 0 für breiig, fest und angedickt = einheitlich; IQA 1 für flüssig = ähnlich).

Die Häufigkeit der Testung zur oralen Boluskontrolle variiert sehr stark (IQA 2 = unterschiedlich).

Die *Überprüfung therapeutischer Maßnahmen* ist in den meisten Kliniken ein häufiger Teil der FEES (58 %) mit geringer Streuung in der Häufigkeit (IQA 0 = einheitlich).

### Befundung und Dokumentation der FEES

Bei Betrachtung der Häufigkeitsverteilung der *beurteilten Parameter* (Vorhandensein Leaking, Vorhandensein Residuen, Menge Residuen, Ort Residuen, Vorhandensein Penetration/Aspiration, Menge Penetration/Aspiration, Zeitpunkt Penetration/Aspiration, Reaktion auf Penetration/Aspiration) zeigt sich eine geringe Streuung in den Häufigkeiten (IQA 0 = einheitlich für 7 der 9 Parameter). Eine regelmäßige Beurteilung („immer“ oder „häufig“) findet für 8 der 9 Parameter in jeweils mindestens 95 % der Fälle statt. Lediglich die Beurteilung der Menge bzw. Häufigkeit von Penetration oder Aspiration wird in 13,83 % der Kliniken nur gelegentlich oder nie betrachtet (IQA 1 = ähnlich).

**Figure 3:**
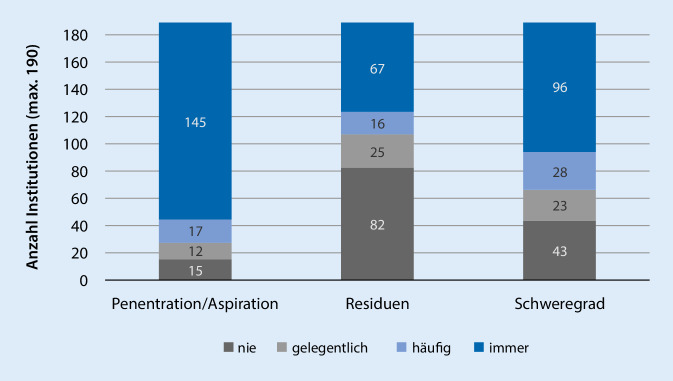

In Abb. 3 ist die Verteilung der Häufigkeiten für die Verwendung von *Scores* für die Beurteilung von Penetration/Aspiration, Residuen und eines Gesamtschweregrades dargestellt. Die Verteilung des Scores für die Penetration und Aspiration zeigt hierfür kaum eine Streuung (IQA 0 = einheitlich). Bei der Verwendung von Scores für die Beurteilung von Residuen und des Schweregrad findet sich eine deutlich erhöhte Streuung (Residuen IQA 3 = unterschiedlich, Schweregrad IQA 2 = ähnlich).

Zur Bewertung von Penetration und Aspiration wird in 93,1 % der Einrichtungen ein veröffentlichter Score eingesetzt. 92,59 % der Institutionen nennen hierfür die Penetrations-Aspirations-Skala (PAS; [8]). Außerdem werden der Münsteraner Dysphagiescore (FEDSS; [14]; 5 Kliniken), die endoskopische Schweregradeinteilung nach Schröter-Morasch ([15]; 5 Kliniken) und der Bogenhauser Dysphagiescore ([13]; 4 Kliniken) angegeben. Residuen werden zu 68,2 % durch einen veröffentlichten Score eingeschätzt. Hierfür werden überwiegend die endoskopische Graduierung von Kelly und Langmore [3] und die Yale Pharyngeal Residue Severity Rating Scale ([16]; jeweils 26 Kliniken) aufgeführt. 14 Kliniken nennen die Beurteilungsskala von Murray [9]. 83,7 % schätzen den Gesamtschweregrad durch einen veröffentlichten Score ein. Hierfür werden überwiegend der Bogenhauser Dysphagiescore ([13]; 57 Kliniken), der FEDSS ([14]; 24 Kliniken) und die endoskopische Schweregradeinteilung neurogener Dysphagien ([17]; 23 Kliniken) beschrieben.

Insgesamt 167 der 190 Kliniken verwenden einen *Protokollbogen* bei der Durchführung der FEES. In 93,41 % handelt es sich hierbei um einen hausinternen Bogen. Zu den verwendeten veröffentlichten Bögen gehören hauptsächlich der Protokollbogen aus dem NOD-Stufenkonzept (Standardisierung des Untersuchungsablaufs bei Neurogener Oropharyngealer Dysphagie; [4]; 5 Kliniken) und der FEDSS [14].

In der Regel werden im abschließenden *Befund* die Hauptbefunde dargestellt (98,4 %), der Schweregrad angegeben (87,4 %) und Diagnostik- und Therapieempfehlungen (95,3 %) sowie Kostempfehlungen (99 %) gegeben. Uneinheitlichkeit besteht bezogen auf die Zuordnung zur Grunderkrankung (61 %), eine syndromale Klassifikation (25,3 %) und die Beschreibung der Pathomechanismen (73,16 %).

### Zusammenhänge

**Figure 4:**
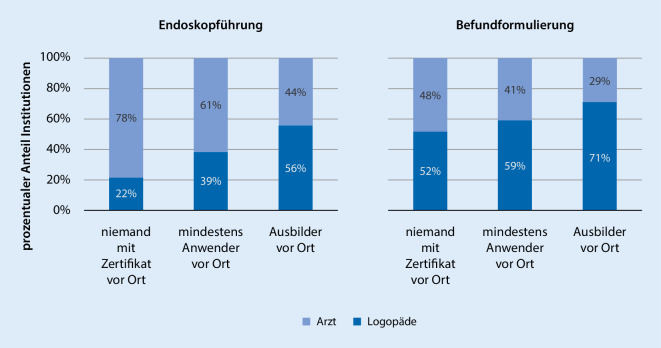

Es zeigt sich ein *Einfluss des Ausbildungsgrads* auf 6 der 22 getesteten abhängigen Variablen, die sich als unterschiedlich in der Durchführung zeigen. Der Ausbildungsgrad wird auf einer 3‑stufigen Rangskala definiert (1 = niemand mit Zertifikat vor Ort, 2 = mindestens ein Anwender vor Ort, 3 = mindestens ein Ausbilder vor Ort). Bezogen auf die Aufgabenverteilung sind sowohl im Bereich der Endoskopführung als auch der Befundformulierung bei zweiseitiger Testung mit der biserialen Rangkorrelation bei einem Wert von −0,284 mit *p* < 0,001 und einem Wert von −0,164 mit *p* = 0,035 signifikante Zusammenhänge zu verzeichnen. In Abb. 4 ist erkennbar, wie mit steigendem Ausbildungsgrad der Anteil der Aufgabenübernahme durch Logopäden steigt und der Anteil der Übernahme durch Ärzte sinkt. Ebenfalls sind signifikante Zusammenhänge zwischen dem Ausbildungsgrad und folgenden Parametern sichtbar: Überprüfung Velumfunktion durch einen trockenen Schluck (0,338, *p* < 0,001, biseriale Rangkorrelation, zweiseitig), Überprüfung von Schlucken aus einem Glas bei angedickter Flüssigkeit (0,270, *p* < 0,001, biseriale Rangkorrelation, zweiseitig), Beschreibung der Pathomechanismen im abschließenden Befund (0,190, *p* = 0,009, biseriale Rangkorrelation, zweiseitig).

Bei 5 getesteten heterogenen Variablen zeigt sich ein *Einfluss durch die Routine* an den Institutionen. Die Routine ist auf einer 3‑stufigen Rangskala in Abhängigkeit von der Anzahl monatlicher FEES definiert: (1) weniger als 10 monatliche FEES, (2) 10 bis 40 monatliche FEES, (3) mehr als 40 monatliche FEES. Es zeigen sich signifikante Zusammenhänge zwischen der Anzahl monatlicher FEES und folgenden Parametern: Überprüfung des Sitzes der Magensonde (0,279, *p* < 001, Spearman-Rangkorrelation, zweiseitig), Durchführung eines trockenen Schluckes zur Überprüfung der Velumfunktion (0,196, *p* = 0,007, biseriale Rangkorrelation, zweiseitig), Überprüfung eines Schluckes aus dem Glas bei angedickter Flüssigkeit (0,151, *p* = 0,044, biseriale Rangkorrelation, zweiseitig), Überprüfung konsekutiver Schlucke bei angedickter Flüssigkeit (0,167, *p* = 0,026, biseriale Rangkorrelation, zweiseitig), Überprüfung der oralen Boluskontrolle (0,195, *p* = 0,007, Spearman-Rangkorrelation, zweiseitig).

**Figure 5:**
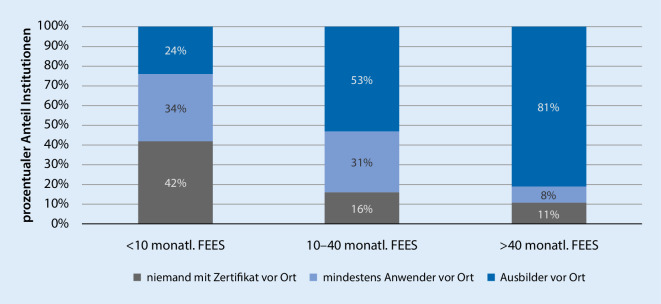

Die zweiseitige Testung mit der Spearman-Rangkorrelation zeigt mit einem Korrelationskoeffizienten von 0,403 mit *p* < 0,001 einen höchstsignifikanten *Zusammenhang zwischen dem Ausbildungsgrad und der Routine der Institutionen*. In Abb. 5 ist erkennbar, dass der Ausbildungsgrad der Institutionen in Abhängigkeit von den monatlich durchgeführten FEES steigt.

## Diskussion

Die FEES gilt als ein unverzichtbares apparatives Instrument im Diagnoseprozess neurogener Dysphagien [2]. Dies unterstreicht die beobachtbar steigende Popularität des Verfahrens, das sich in einer wachsenden Anzahl an durchführenden Institutionen und einer Vielzahl an aktuell Auszubildenden in dem Bereich zeigt. Das steigende Ansehen der FEES ist zeitlich stark mit der Einführung des Ausbildungscurriculums verknüpft.

Vergleichbar mit den Ergebnissen der FEES-Registerstudie [18] wird durch diese Befragung die hohe Involvierung der Berufsgruppe Logopädie an der FEES-Untersuchung dargelegt. Die Beobachtung der Registerstudie kann durch die Betrachtung der Verteilung der Verantwortlichkeit von Ärzten und Logopäden auf die einzelnen Aufgabenbereiche erweitert werden: In der FEES-Registerstudie wurde aufgeschlüsselt, zu welchen Anteilen die Berufsgruppen an der Untersuchung beteiligt sind. In 41,2 % war dies der Logopäde alleine. Dies reflektiert sich in unserer Beobachtung, dass in 38,3 % die Endoskopführung ebenfalls durch Logopäden durchgeführt wird. Diese Aufgabenteilung ist jedoch bundesweit heterogen und wird in unseren Daten zumindest partiell durch das Vorhandensein eines curricularen Anwenders oder Ausbilders erklärt: Je höher der curriculare Ausbildungsstand, desto höher der Anteil an endoskopführenden Logopäden. Hier könnte ein kausaler Zusammenhang postuliert werde, dass nämlich mit Einführung der curricularen FEES auch ein Transfer von Verantwortlichkeiten stattgefunden hat. Dennoch sind Ärzte in unserer Studie auch weiterhin in höherem Maße für die praktische Durchführung der Untersuchung und die Logopäden für die Interpretation der Ergebnisse zuständig, was besonders an der Kostformempfehlung deutlich wird. Aus der FEES-Registerstudie lässt sich zudem ein erhöhter Anteil sehr erfahrener Anwender bei potenziell komplikationsträchtigen Patienten ableiten, was verdeutlicht, dass die Rollenzuweisung oft nicht strukturell starr, sondern situationsabhängig sein dürfte.

Weiterhin führen die Zentren, die einen hohen Bezug zum Curriculum aufweisen (Ausbilder vor Ort) deutlich mehr FEES durch als andere. Hier lässt sich nur schwer eine kausale Richtung aufzeigen: Es wäre sowohl denkbar, dass Ausbilder nur an Zentren generiert werden, die über die notwendige Zahl an jährlich durchgeführten FEES verfügen, als auch dass FEES-Ausbilder vor Ort die Kapazitäten steigern sowie auch die Indikationsstellung beeinflussen. Unsere Daten können dies nicht suffizient trennen, mutmaßlich treffen beide Annahmen in unterschiedlichen Wichtungen zu.

Zentren mit hohem curricularem Bezug führen zudem mehr Schritte der Untersuchung durch und formulieren detailliertere Befunde, sowohl was die Beurteilung schluckrelevanter Strukturen als auch der applizierten Konsistenzen und der mutmaßlichen Pathophysiologie angeht. Wir sehen hier einen Einfluss des Curriculums, welches ja auch einen intensiven evidenzbasierten theoretischen Ausbildungsanteil vorsieht, auf den Umfang und damit die Qualität der Untersuchung sowie auch auf die Befunde. Diese bewegen sich mit zunehmender Assoziation an das Curriculum möglicherweise weg vom rein deskriptiven Beschreiben der augenfälligsten Befunde hin zu einer umfassenden Beurteilung der beobachteten Schluckanatomie und -physiologie.

Zu einer völligen Harmonisierung oder unkritischen Standardisierung hat die Implementation des FEES-Curriculums jedoch nicht geführt. Die Durchführung der Untersuchung weist trotz der Orientierung am Standardprotokoll nach Langmore [3] an einigen Stellen Unterschiedlichkeiten auf. Teilweise handelt es sich bei den heterogenen Parametern um solche, deren Anwendung vor dem Hintergrund einer gewünschten Ökonomie der Untersuchung nur im Bedarfsfall notwendig erscheint. Kritisch anzumerken ist zumindest das teilweise Auslassen der anatomisch-physiologischen Untersuchung, welches die Vollständigkeit der Diagnostik gefährdet. Bei Durchführung dieses Untersuchungsblocks findet sich außerdem eine für die Interpretation der Symptome unzureichende Beurteilungshäufigkeit notwendiger Funktionen. Aus unseren Daten kann leider nicht abgeleitet werden, in welchen Strukturen dies gehäuft vorkommt, da nur die wenigsten Fragebögen einem einzelnen Setting (Intensiv/Stroke/Normalstation) oder einer einzelnen Fachrichtung (Neuro/Geri/Innere/HNO) zuzuordnen waren. Es könnte jedoch spekuliert werden, dass die FEES auf Intensivstationen und Stroke-Units, wo schwer betroffene Patientenpopulationen mit reduzierter Fähigkeit zur Kooperation gehäuft auftreten, hier möglicherweise verstärkt beiträgt.

Zusätzlich fehlt im Bereich der Schluckversuche teilweise eine Vereinheitlichung der verabreichten Konsistenzen und Nahrungsmittel. Ob hier die „International Dysphagia Diet Standardisation Initiative“ (IDDSI, https://iddsi.org) hilfreich sein kann, bleibt abzuwarten. Besonders in Bezug auf das Scoring schluckrelevanter Parameter herrscht zudem Standardisierungsbedarf. Dieser ist für eine valide und reliable Befunderstellung und bessere Vergleichbarkeit dringend erforderlich.

In der Summe legen unsere Daten nahe, dass das FEES-Curriculum mit einer Entlastung der ärztlichen Berufe assoziiert ist bei einer gleichzeitigen quantitativen Ausweitung dieser wichtigen Diagnostik verbunden mit detaillierteren und pathophysiologisch orientierten Befunden. Damit erfüllt es die wesentlichen an das Curriculum gestellten Anforderungen.

Es muss als wesentliche Limitation unserer Erhebung darauf hingewiesen werden, dass in der vorliegenden Studie überwiegend ein exploratives Vorgehen Anwendung fand, um einen Überblick über den aktuellen Stand der Standards bei der Anwendung der Diagnostik zu erhalten und weitere Fragestellungen abzuleiten. Vor dem Hintergrund der Studienergebnisse ist eine tiefergehende Betrachtung einzelner Parameter sinnvoll. Besonders im Bereich des Scorings sind weitergehende Studien zum Vergleich einzelner Scores nötig, bevor eine einheitliche oder gar verbindliche Festlegung zu rechtfertigen wäre. Generell kann das FEES-Curriculum aber in Zukunft als Vehikel zur weiteren Vereinheitlichung von als sinnvoll erachteten Standardparametern dienen, ohne dass dem Therapeuten vor Ort die Freiheit in der individuellen Befunderhebung entzogen würde. Wünschenswert wäre vor diesem Hintergrund möglicherweise ein auf das Curriculum zugeschnittener veröffentlichter Protokollbogen, dessen Anwendung eine vollständige Erhebung des endoskopischen Befundes sichern könnte und der als Grundlage für Qualitätssicherungsmaßnahmen und/oder multizentrische Studien Verwendung finden könnte.

## Fazit für die Praxis

Die Einführung des Curriculums hat zu einer inhaltlichen und quantitativen Aufwertung der FEES und zu einer Stärkung der Logopädie als durchführende Berufsgruppe geführt, wodurch eine bessere, wenn auch nicht flächendeckende Verbreitung des Verfahrens ermöglicht wurde. Die Durchführung der FEES in Deutschland weist einen in wesentlichen Aspekten homogenen Ablauf der Untersuchung und Befundung auf. Gleichzeitig wird Potenzial an weiterer Vereinheitlichung erkennbar. Das FEES-Curriculum könnte als Werkzeug zur weiteren Standardisierung verwendet werden.
